# Integrated framework for targeting dynamic penicillin-binding protein 2a via ensemble structural biology and deep generative modeling

**DOI:** 10.3389/fmicb.2026.1811950

**Published:** 2026-05-11

**Authors:** Tianshu Pang, Xiangyu Zhang, Yuan Zhou, Jin Yang

**Affiliations:** Department of General Surgery, Sir Run Run Shaw Hospital, Zhejiang University School of Medicine,, Hangzhou, China

**Keywords:** antibacterial discovery, deep generative modeling, ensemble docking, MD, MRSA, PBP2a

## Abstract

*Methicillin-resistant Staphylococcus aureus (MRSA)* remains a major cause of morbidity and mortality worldwide, largely due to penicillin-binding protein 2a (PBP2A), a low-affinity transpeptidase encoded by mecA. To address the challenge of this highly flexible target, we developed an integrated discovery framework combining conformational ensemble analysis with deep generative modeling. Extensive molecular dynamics simulations were used to map the dynamic landscape of PBP2A and identify cryptic binding pockets. A target-conditioned generative workflow then explored these states to produce novel candidate scaffolds, followed by multi-objective optimization and structural prioritization. Among the generated molecules, Compound 1 showed a conserved binding mode, sustained stability over 1000 ns MD simulations, and more favorable MMGBSA binding energies than native ligands. Whole-cell assays showed dose-dependent growth inhibition against *S. aureus* ATCC 29213 and *MRSA* ATCC 43300, with stronger activity after 24 h exposure. Additional propidium iodide staining revealed a concentration-dependent increase in PI uptake in both strains, consistent with compound-induced envelope perturbation. These findings support Compound 1 as an antibacterial lead emerging from a PBP2A-guided discovery strategy, while direct target engagement remains to be established.

## 1 Introduction

The global escalation of antimicrobial resistance represents a formidable challenge to modern medicine (Patra et al., 2025). Among the most dangerous pathogens methicillin resistant *Staphylococcus aureus* remains a primary cause of high morbidity and mortality worldwide due to its resistance to almost all beta lactam antibiotics (Thacharodi et al., 2026). This resistance is fundamentally mediated by the expression of penicillin binding protein 2a which is a transpeptidase encoded by the mecA gene (Shalaby et al., 2020). Unlike essential proteins in susceptible strains this protein possesses an extremely low affinity for antibiotics and allows cell wall biosynthesis to persist even in the presence of lethal drug concentrations (Dabhi et al., 2024).

The structural basis of this resistance lies in the highly dynamic and closed active site of the protein (Xu et al., 2024; Song et al., 2025; Chu et al., 2025). Crystallographic studies have revealed that the catalytic pocket of the protein is sterically occluded to prevent the entry of traditional antibiotics (Matoba et al., 2006). Access to this site is strictly regulated by an allosteric site located approximately 60 angstroms away from the catalytic center (McCullagh et al., 2024). The binding of a ligand to this distal site triggers a long range conformational change that unlocks the active site (Zarifi et al., 2025). Consequently traditional structure based drug design which relies on static snapshots from X ray crystallography often fails to capture the transient druggable states of this highly flexible protein (Cao et al., 2024).

To overcome the limitations of static models the integration of conformational ensembles has emerged as a superior paradigm (Kopylov et al., 2025). By employing extensive molecular dynamics simulations and enhanced sampling techniques researchers can map the structural plasticity of the target (Bernardi et al., 2015). These ensembles represent a statistical distribution of the structural states to reveal cryptic pockets and intermediate open configurations that are invisible in static structures (Aranganathan et al., 2025). Targeting a conformational ensemble rather than a single structure allows for the identification of inhibitors that can either stabilize the inactive state or exploit transiently exposed motifs (Nussinov et al., 2023).

Parallel to advances in structural biology deep learning based generative models have revolutionized the expansion of the accessible chemical space. Conventional virtual screening is often restricted to predefined libraries of manageable size while generative models enable the *de novo* design of novel molecular scaffolds with optimized pharmacological properties (Corrêa Veríssimo et al., 2025). By conditioning these generative models on the structural features derived from the conformational ensembles it is possible to tailor molecules that satisfy the complex geometric and electrostatic requirements of dynamic binding sites (Brownless et al., 2025).

**Figure 1:**
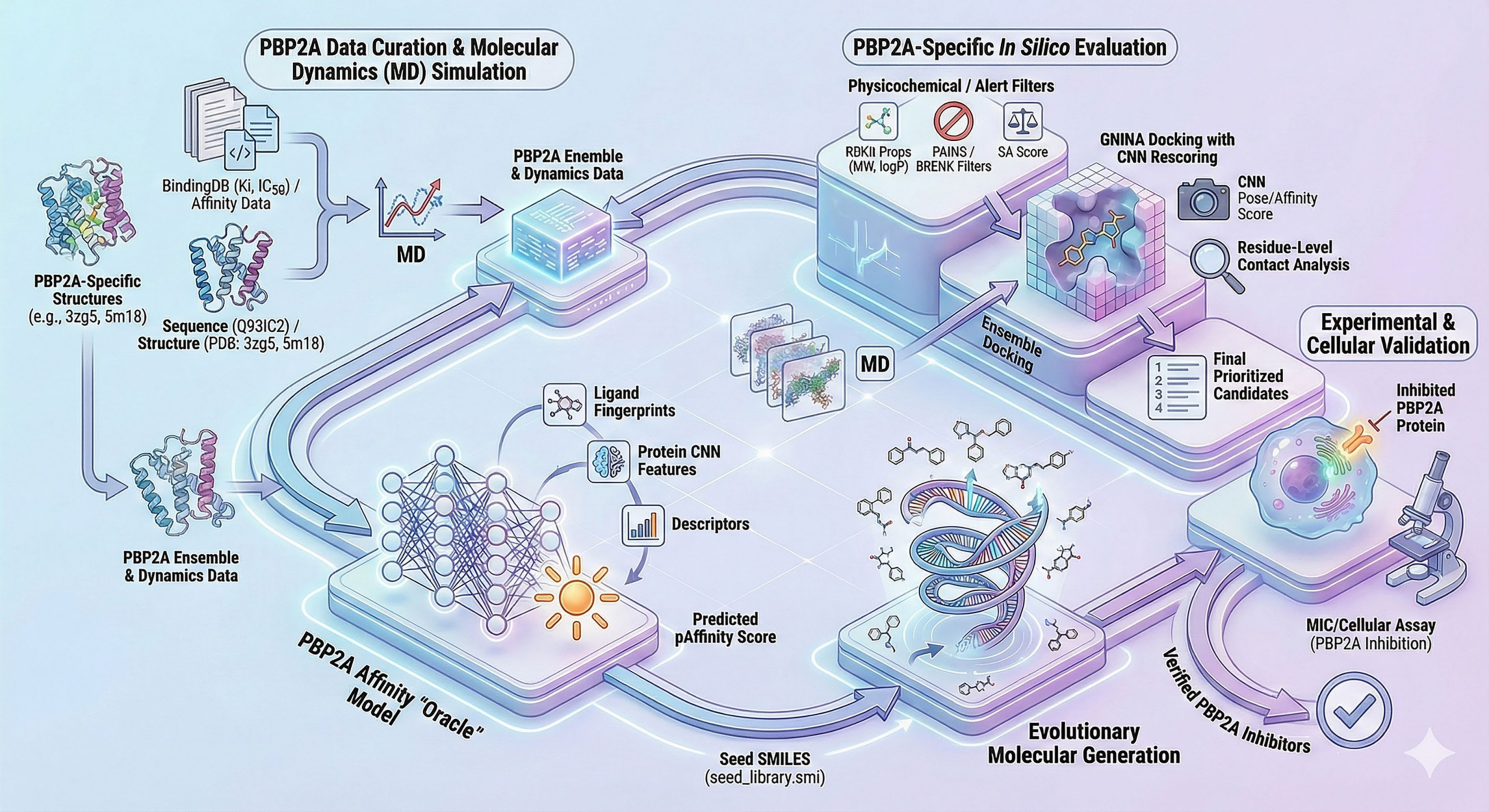
Research flow chart.

This study proposes a framework that integrates ensemble based structural biology with deep generative modeling to develop next generation inhibitors. We first characterize the conformational landscape of the protein to identify key functional states and transient pockets. Subsequently we utilize a target conditioned generative model to sample the chemical space corresponding to these dynamic states. This approach identifies novel lead compounds with potential activity against resistant strains and provides a generalizable workflow for targeting other highly flexible proteins. The specific workflow of this study is illustrated in Figure 1.

## 2 Methods

### 2.1 Data and preprocessing

Binding affinity data were retrieved from BindingDB^1^ and filtered to the target of interest. Inhibition constant (Ki), half-maximal inhibitory concentration (IC50), dissociation constant (Kd), and half-maximal effective concentration (EC50) values (nM) were cleaned, the first available quantitative entry was selected per record, and activities were converted to pAffinity (`9 − log10(nM)`, clipped to [4,10]) with Simplified Molecular Input Line Entry System (SMILES) deduplication. The target protein sequence was provided as a FASTA-formatted sequence when available.

### 2.2 Affinity prediction (oracle) model

Ligands were encoded using 2048-bit Morgan fingerprints (radius = 2) together with six normalized physicochemical descriptors, including molecular weight (MW), LogP, number of hydrogen-bond donors (HBD), number of hydrogen-bond acceptors (HBA), topological polar surface area (TPSA), and number of rotatable bonds (Zhou and Skolnick, 2024). The target protein sequence was represented at the amino-acid level using a vocabulary of the 20 canonical residues, integer-encoded, and zero-padded/truncated to a fixed length of 1,000 residues. Sequence tokens were embedded into a 64-dimensional space and processed by a one-dimensional convolutional neural network composed of three convolutional blocks with channel dimensions of 64 → 32, 32 → 64, and 64 → 128 and kernel sizes of 12, 8, and 6, respectively. Max-pooling was applied after the first two convolutional layers, and the final sequence representation was compressed using adaptive max-pooling to generate a fixed-length protein embedding.

The protein embedding was concatenated with the ligand fingerprint and descriptor features and passed to a multilayer perceptron with hidden dimensions of 1,024, 512, and 128. ReLU activation was used throughout, with batch normalization and dropout (0.4) applied in the first two fully connected hidden layers, followed by a single scalar regression output for affinity prediction. The complete input dimension to the fusion network was 2,182, comprising the ligand fingerprint, physicochemical descriptors, and learned protein-sequence embedding (Tasnim et al., 2024).

No external pre-training was used. The affinity predictor was trained *de novo* on the curated binding-affinity dataset using the Adam optimizer with a learning rate of 0.001, weight decay of 1 × 10^-5, and batch size of 32. A 90:10 train/validation split was used for model development, and model performance was monitored using the coefficient of determination (R^2^). To improve robustness, three independently initialized DeepDTA-style models were trained adaptively (Öztürk et al., 2018). For each model, training was repeated for up to seven attempts, with up to 200 epochs per attempt. Validation R^2^ was evaluated periodically, learning-rate reduction on plateau was applied, and early stopping was triggered when performance no longer improved. A minimum validation threshold of R^2^ ≥ 0.70 was used as the acceptance criterion for the deep model.

When the deep-learning model failed to satisfy the predefined validation threshold, a tree-based scikit-learn fallback oracle was used to maintain the stability of the downstream generative workflow. In this fallback stage, Extra Trees, Random Forest, and Gradient Boosting regressors were evaluated, and the best-performing model was selected according to validation R^2^. This hybrid strategy ensured that the subsequent molecular generation and prioritization steps were driven by the most reliable available affinity estimator under the current data regime.

### 2.3 Molecule generation and filtering

Candidate molecules were generated using an evolutionary strategy initialized from seed compounds. New structures were created through Breaking of Retrosynthetically Interesting Chemical Substructures (BRICS)-based fragment recombination and fragment-attachment mutations (Wang and Wang, 2025). Candidates were scored by a multi-objective function combining predicted affinity, quantitative estimate of drug-likeness (QED), synthetic-accessibility (SA) proxy, and novelty, while applying hard filters to remove compounds with structural alerts from pan-assay interference compounds (PAINS), Brenk alerts (BRENK), or National Institutes of Health (NIH) filters, as well as out-of-range physicochemical profiles.

### 2.4 Docking and post-generation evaluation

Structure-based evaluation was carried out with GNINA (McNutt et al., 2025). Native ligands in protein–ligand complexes were used to define docking boxes (Huang et al., 2009; Mahasenan et al., 2017); receptors were prepared by removing the native ligand and converting to docking format. Ligands were conformer-generated and optimized, then docked with GNINA using convolutional neural network (CNN) rescoring. Final prioritization integrated docking scores (affinity and CNN metrics) with property/alert filters, and residue-level contact analysis was used to summarize key interactions.

### 2.5 Molecular dynamics (MD) simulation

Molecular dynamics simulations of the PBP2A complexes were performed for 1,000 ns at 300 K using the Desmond package and the Optimized Potentials for Liquid Simulations 4 (OPLS4) force field. Each system was solvated in a three-point transferable intermolecular potential (TIP3P) water box with 0.15 M NaCl and equilibrated under constant number of particles, pressure, and temperature (NPT) conditions before the production phase. Structural stability was monitored by calculating the root mean square deviation (RMSD) of the protein backbone and ligand atoms. Binding free energies were quantified using the MMGBSA approach on the final 20 ns of the trajectory to determine the thermodynamic stability of the predicted inhibitors.

### 2.6 Minimum inhibitory concentration (MIC) assay

The antibacterial activity of Compound 1 was evaluated against *Staphylococcus aureus* ATCC 29213 and *S. aureus* ATCC 43300 by broth microdilution in cation-adjusted Mueller-Hinton broth (CAMHB) using sterile 96-well microtiter plates. ATCC 29213 was used as a methicillin-susceptible reference strain, whereas ATCC 43300 was used as a methicillin-resistant strain carrying the PBP2a-associated resistance determinant. A fresh culture was prepared from an overnight tryptic soy agar plate, and the inoculum was adjusted and diluted in CAMHB to obtain the required final cell density in each well. Compound 1 was prepared as a stock solution in dimethyl sulfoxide (DMSO) and serially diluted two-fold in CAMHB to generate the full assay concentration panel. Wells were prepared to a final volume of 100 μL and contained a constant final DMSO concentration of 0.5% (v/v) across all test and vehicle-control wells. Each concentration was tested in six technical replicates per plate. Growth controls (no compound, with 0.5% DMSO) and sterility controls (medium only) were included on every plate. Plates were incubated statically at 35 °C under aerobic conditions, and bacterial growth was quantified by measuring OD_600_ at both 18 h and 24 h for Compound 1. For ATCC 43300, ceftaroline was included as a positive control and was evaluated using the same broth microdilution format. MIC was operationally defined as the lowest concentration that resulted in complete inhibition of growth, corresponding to the absence of visible turbidity and an OD_600_ value less than or equal to the sterility control mean plus three standard deviations (blank mean + 3 SD). All assays were performed in three independent experiments on separate days.

## 3 Results

### 3.1 Evaluation of model training stability and evolutionary optimization dynamics

**Figure 2:**
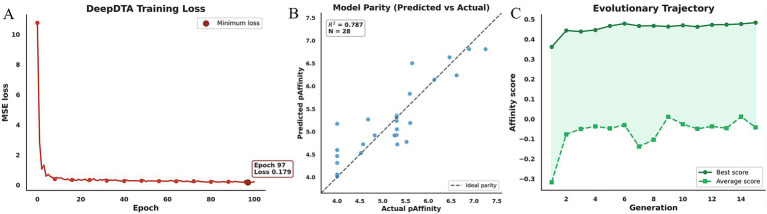
Evaluation of DeepDTA model performance and evolutionary optimization dynamics. **(A)** Training loss curves showing a rapid initial convergence followed by a stable plateau. The minimal standard deviation (SD) over the final 10 epochs indicates a well-controlled training process and high reproducibility of the SAR signals. **(B)** Parity plot illustrating the correlation between predicted and experimental binding affinities (R-squared = 0.787). The alignment of data points along the diagonal demonstrates the model’s robust ranking capability, while the observed dispersion highlights the predictive uncertainty addressed by subsequent docking constraints. **(C)** Evolutionary search trajectories of the best and average fitness scores across generations. The steady rise of the best score reflects the continuous refinement of elite candidates, whereas the negative fluctuations in the average score (driven by a penalty of −1 for non-compliant samples) underscore the impact of stringent hard filters on the population’s distribution within the feasible chemical space.

The training process and predictive performance of the DeepDTA model were first evaluated to ensure the reliability of the molecular generation pipeline. As shown in Figure 2A, the training loss curve exhibits a rapid initial decline, indicating that the model effectively captured the primary structure–activity relationship (SAR) signals from the dataset. The subsequent stabilization of the loss within a narrow window, characterized by a minimal standard deviation (SD) over the final ten epochs, suggests that the optimization reached a convergence zone dominated by representation limits rather than stochastic noise. The absence of significant fluctuations or late-stage rebounds underscores the controllability and reproducibility of the deep learning model’s training under the current settings. This predictive foundation is further supported by the parity plot in Figure 2B, where the data points generally align along the diagonal with an R-squared value of 0.787. Although this correlation demonstrates a robust ranking capability suitable for candidate screening, the observed dispersion highlights inherent prediction uncertainties. Within the evolutionary search framework, such uncertainty necessitates a balanced reliance on diversity and novelty terms to prevent the search from being trapped in local optima or noise-driven biased structures. Consequently, we integrated molecular docking as an external geometric-energetic constraint to reconcile statistical predictions with physical binding interactions, thereby minimizing potential false positives.

To facilitate ensemble-based molecular generation, MD simulations were performed on both 3ZG5 and 5 M18 crystal structures, followed by clustering each trajectory into ten representative conformations. This process yielded a total of 20 protein-inhibitor complex models that served as the structural basis for the generative workflow. The dynamics of this optimization are further illustrated by the evolutionary search trajectories in Figure 2C, where the gap between the maximum and average fitness scores serves as an indirect metric for the population distribution within the feasible chemical space. We observed a characteristic hard-constrained evolution phenomenon where the best scores improved consistently as elite individuals were refined while the average scores frequently dropped into negative values. This discrepancy stemmed from the application of stringent filters including synthetic accessibility (SA), toxicity alerts, and physicochemical constraints that penalized non-compliant candidates with a score of negative one. These results demonstrate that the generative workflow entails extensive structural exploration where elite preservation and regeneration mechanisms successfully and steadily advance the upper bound of molecular quality even though a significant portion of variants falls into unacceptable regions. After preprocessing and SMILES deduplication, 274 nonredundant ligand-activity records were retained for model training. The generative search was initialized from 6 seed compounds and produced 120 unique molecules. Among these, 82 remained in the feasible scoring region (Ensemble Score > − 1), whereas 38 received hard-filter penalties (Ensemble Score = −1). These statistics quantitatively illustrate the balance between chemical exploration and constraint enforcement in the generative workflow.

### 3.2 Multi-objective optimization and characterization of the generated chemical space

**Figure 3:**
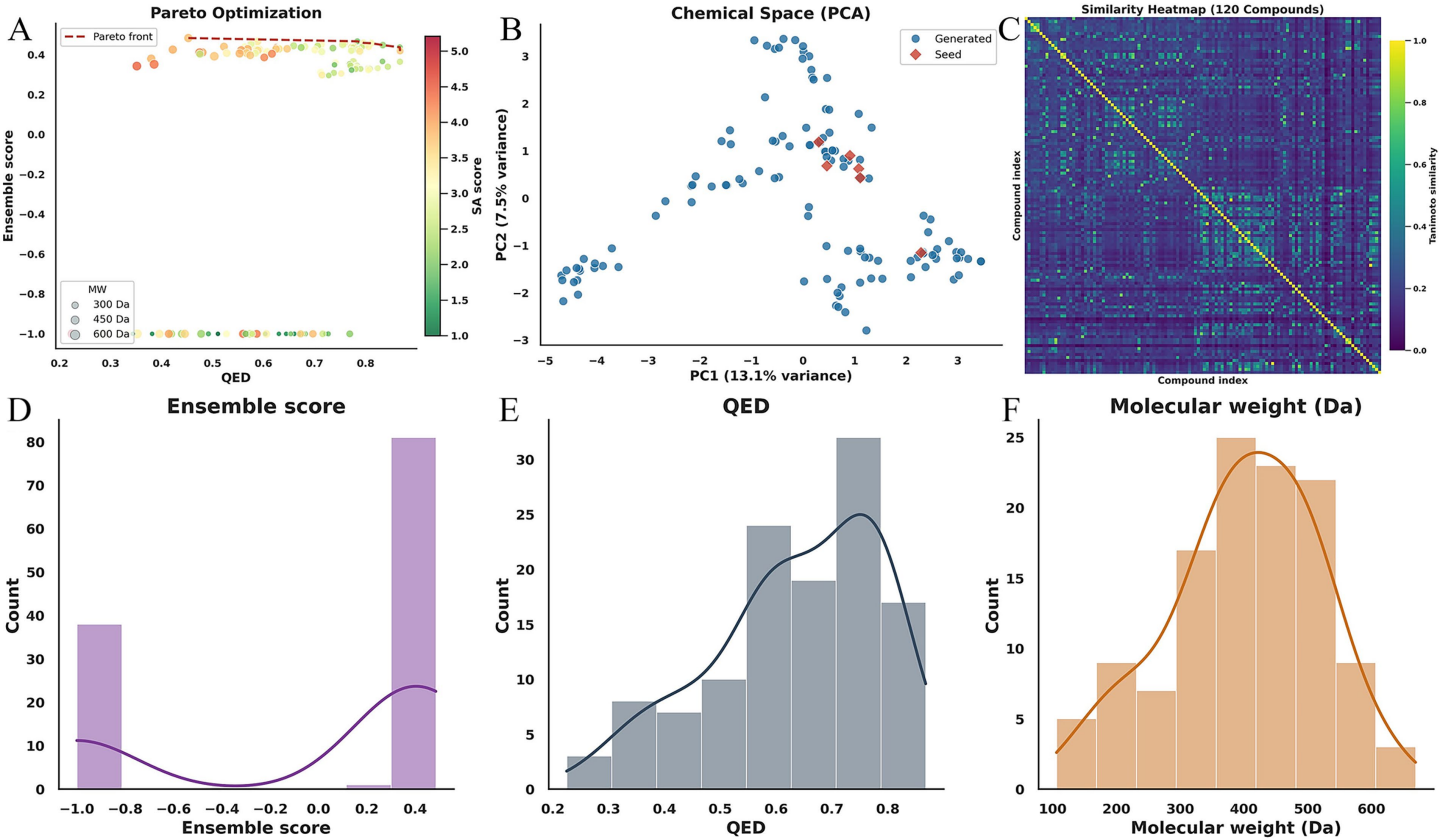
Multi-objective optimization, chemical space exploration, and library diversity. **(A)** Pareto frontier of QED versus ensemble score, where color and size represent SA and MW, respectively. The red dashed line indicates the Pareto-optimal skyline. **(B)** PCA-based chemical space visualization comparing generated molecules (*n =* 120) with seeds (*n =* 6). Distance-based metrics (centroid distance = 1.174) underscore the model’s ability to transcend the seed structural neighborhood. **(C)** Pairwise similarity analysis, where the heatmap and cluster metrics (median similarity = 0.172; 33 clusters at 0.6 threshold) demonstrate high library diversity and scaffold variety. **(D–F)** Property distribution profiles for ensemble score, QED, and MW. The distributions reveal the impact of hard filters (ensemble score = −1 peak) and the successful maintenance of drug-like properties despite the optimization pressure for higher binding affinity.

The balance between drug-likeness, binding affinity, and synthetic feasibility was evaluated through a multi-objective optimization analysis. As illustrated in the Pareto frontier plot (Figure 3A), we mapped the relationship between QED (x-axis) and the Ensemble Score (y-axis), with points color-coded by synthetic accessibility (SA) and sized by molecular weight (MW). A distinct hard-filtering belt is observed at Ensemble Score = −1, representing candidates that failed to meet the stringent prior constraints, thereby compressing the visual proportion of the feasible region. The red dashed line denotes the Pareto-optimal frontier (skyline), representing the upper bound of achievable performance where one objective cannot be improved without sacrificing another. Ideally, the most promising lead candidates are situated toward the upper-right quadrant, achieving high QED and Ensemble Score while maintaining acceptable SA levels.

To assess the structural novelty and the extent of chemical space exploration, we performed a Principal Component Analysis (PCA) on the molecular fingerprints of the generated library (*n =* 120) relative to the seed molecules (*n =* 6) (Figure 3B). Given the high dimensionality and sparsity of the fingerprint space, the cumulative explained variance of the first two components was 20.5% (PC1: 13.1%; PC2: 7.5%), which is typical for diverse chemical datasets. Therefore, distance-based metrics provided a more robust measure of exploration: the centroid distance between the generated and seed populations reached 1.174, with a median distance to the nearest seed of 1.176 and a 90th percentile (P90) of 4.952. These results indicate that the generative model did not merely perform local refinements but successfully navigated into novel chemical territories far beyond the structural neighborhood of the starting seeds.

The internal diversity of the generated library was further quantified through pairwise Tanimoto similarity analysis (Figure 3C). The distribution is characterized by a low median similarity of 0.172 and a P90 of 0.333, with only 0.2% of pairs exceeding a similarity threshold of 0.8. Cluster analysis at a 0.6 similarity threshold revealed 33 distinct connected components, with the largest cluster containing only 20 molecules. This multi-family, small-cluster architecture suggests that the library is not dominated by a single scaffold, thereby mitigating the risk of systematic failure arising from model bias or specific docking inaccuracies.

Finally, the property distributions shown in Figures 3D–F demonstrate that under the joint influence of hard constraints and scoring functions, the search process did not drift toward extreme regions of excessive molecular weight or lipophilicity. The peak at −1 in the Ensemble Score distribution serves as a direct indicator of the generative operator’s out-of-bounds exploration intensity, while the positive distribution peaks at approximately 0.48, representing the practical performance ceiling under the current configuration. The joint distribution of QED and MW highlights a fundamental medicinal chemistry tension: while increasing the molecular surface area often improves binding interactions, it simultaneously elevates MW and potential absorption, distribution, metabolism, excretion, and toxicity (ADMET) risks. Our workflow successfully navigated this trade-off, identifying candidates that maximize activity while remaining within a developable physicochemical window.

### 3.3 Drug-likeness assessment, structural characterization, and feature importance analysis

**Figure 4:**
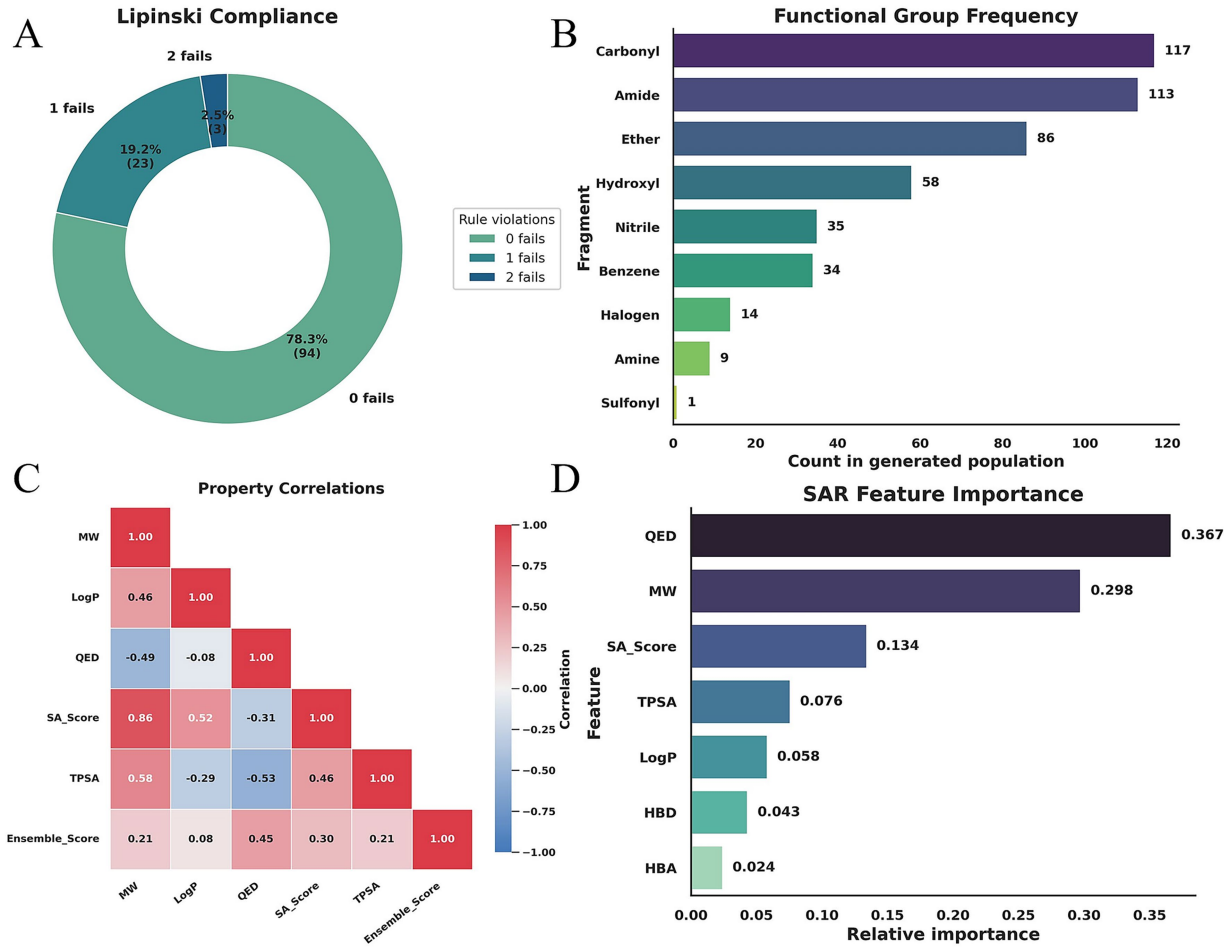
Drug-likeness assessment, fragment analysis, and feature importance. **(A)** Distribution of Lipinski Rule of Five violations and a statistical breakdown of hard-filtering reasons, showing that toxicity alerts and molecular weight limits are the primary constraints on the feasible chemical space. **(B)** Frequency of functional groups and fragments, highlighting the high prevalence of hydrogen-bond donors/acceptors and the model’s strategic use of small polar moieties like nitriles. **(C)** Property correlation matrix illustrating the strong dependencies between molecular weight, synthetic complexity, and overall drug-likeness. **(D)** Feature importance analysis based on a Random Forest model, identifying QED and MW as the primary determinants driving the Ensemble Score optimization.

To evaluate the potential of the generated candidates as oral drugs, we first analyzed the distribution of Lipinski Rule of Five (Ro5) violations. As shown in Figure 4A, the majority of the library exhibits zero violations, indicating that the generative process effectively remained within the boundaries of acceptable drug-likeness. This coarse-grained assessment is further refined by a detailed breakdown of the Ensemble Score = −1 hard-filtering events. Our analysis reveals that these exclusions were predominantly driven by Toxic Alerts, which triggered 23 times due to the presence of problematic moieties identified by PAINS, BRENK, or NIH filters. Additionally, 15 candidates were excluded for having a MW below 250, suggesting a lack of sufficient interaction surfaces. Other minor factors included excessive hydrophilicity (LogP < −0.5) or lipophilicity (LogP > 5.0), and overall poor QED scores. Notably, no molecules were rejected based on the SA Score limit of 6.5, confirming that the generated candidates remained synthetically accessible. These statistics suggest that the evolutionary operator frequently explores structural variants that are either chemically reactive or too small, highlighting an opportunity to improve yield by refining initialization strategies or constraining generative operators within optimal size and safety windows.

The structural composition of the library was further characterized by the frequency of specific functional groups and fragments in Figure 4B. The high prevalence of Carbonyl (117), Amide (113), Ether (86), and Hydroxyl (58) groups provides a rich reservoir for diverse hydrogen-bonding interactions. The frequent appearance of Nitrile (35) groups indicates the model’s preference for small, high-dipole fragments that enhance directional interactions without significantly increasing molecular weight. Furthermore, the presence of Benzene (34) and Halogen (14) atoms provides the necessary hydrophobicity and shape complementarity for target binding.

The interdependencies between these physicochemical properties were examined via a correlation matrix in Figure 4C. We observed a strong positive correlation between MW and the SA Score (0.862), which aligns with the intuition that larger molecules typically possess higher synthetic complexity. MW also showed moderate correlations with TPSA (0.582) and LogP (0.457). Conversely, QED exhibited a notable negative correlation with both TPSA (−0.532) and MW (−0.494), suggesting that increases in polarity or size may negatively impact overall drug-likeness scores. It is important to note that since these correlations include samples that triggered the Ensemble Score = −1 penalty, these outliers may amplify certain statistical relationships, necessitating a more granular analysis of the high-scoring subset to identify fine-tuned drivers of activity.

Finally, we employed a Random Forest model to identify the primary drivers of the Ensemble Score, as illustrated in Figure 4D. QED (0.367) and MW (0.298) emerged as the most influential features, reflecting the scoring function’s design where QED is a direct component and MW is coupled with both feasible region constraints and binding affinity. The SA Score (0.134) and TPSA (0.076) occupied the second tier of importance, indicating that synthetic accessibility and polarity are key factors in differentiating candidates within high-scoring regions. However, because the Ensemble Score = −1 threshold creates a strong training signal, the model may overemphasize properties that trigger hard filters. Therefore, conducting a subsequent importance analysis on the valid subset where the score is greater than zero would be instrumental in identifying the fine-grained drivers within the feasible chemical space.

### 3.4 Molecular docking validation and binding interaction analysis

Molecular docking was employed as a structural constraint to transition from statistical proxies of activity to a physically grounded evaluation of three-dimensional geometric and energetic consistency. The binding affinities across the library exhibited a mean of −7.171 with a standard deviation of 1.460. The optimal binding affinity reached −9.42, indicating a strong interaction, while the results generally centered around −7, suggesting that the generated molecules occupy a moderate-to-strong binding range. A detailed analysis grouped by the receptor targets, 3ZG5 and 5 M18, further confirms this performance. For 3ZG5, the mean affinity was −7.417 (minimum −9.42), while 5 M18 showed a mean of −6.927 (minimum −9.29). For structure-based prioritization, 20 receptor conformations were used for GNINA docking, comprising 10 representative structures derived from 3ZG5 and 10 from 5 M18. A total of 40 candidates entered the coarse docking stage, after which 30 docking entries were retained for refinement. Following aggregation, these refinement-stage entries corresponded to 14 unique SMILES. Final post-docking filtering, integrating docking performance with QED, SA score, and toxicity constraints, yielded 3 lead candidates for detailed interaction analysis and prioritization.

**Figure 5:**
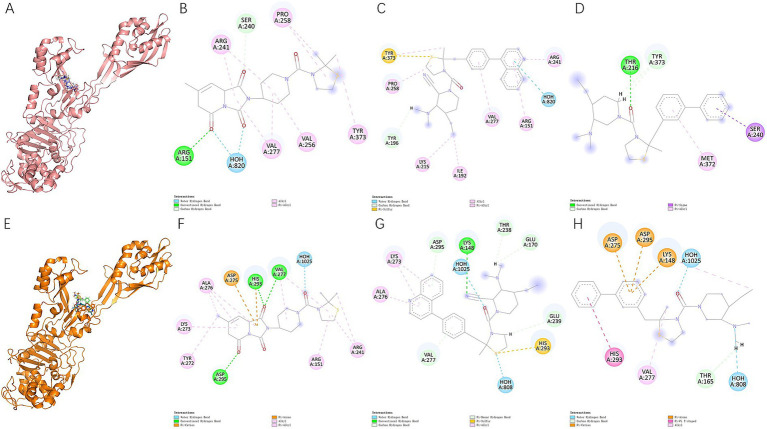
Molecular docking enrichment and binding interaction analysis for lead candidates. **(A)** Complex models of Compound 1, Compound 2, and Compound 3 bound to the 3ZG5 receptor. **(B–D)** Detailed 2D/3D interaction diagrams for Compounds 1, 2, and 3 within the 3ZG5 binding pocket. **(E)** Complex models of the three candidates bound to the 5 M18 receptor. **(F–H)** Detailed interaction networks for Compounds 1, 2, and 3 within the 5 M18 binding pocket. The analysis highlights the conserved residues for Compound 1 (ARG-241, ARG-151, VAL-277, and TYR-373) across both models, contrasting with the low or zero residue overlap observed for Compound 2 and Compound 3.

The enrichment of docking results and the specific binding modes for the top three candidates are illustrated in Figure 5. We focused on the structural robustness of Compound 1, Compound 2, and Compound 3 across both target receptors. Figure 5A displays the complex models of these three compounds bound to 3ZG5, with their corresponding interaction networks detailed in Figures 4B–D. Similarly, Figure 5E presents the complex models for the 5 M18 receptor, with individual interaction analyses for the three compounds shown in Figures 4F–H.

A comparative analysis of the binding poses revealed significant differences in interaction consistency between the two protein models. Compound 1 demonstrated a highly conserved binding mode, sharing four key residues—ARG-241, ARG-151, VAL-277, and TYR-373—across both 3ZG5 and 5 M18 complex models. In stark contrast, Compound 2 exhibited poor conservation, sharing only a single residue (VAL-277), while Compound 3 showed no overlapping residues between the two receptor environments. This lack of residue-level consistency suggests that the binding orientations of Compound 2 and Compound 3 may be highly sensitive to minor conformational changes in the protein or represent less stable binding modes. Consequently, Compound 1 was identified as the most promising lead for further development, while Compound 2 and Compound 3 were excluded from subsequent analyses. To contextualize the performance of the proposed workflow, we performed internal comparative analyses against several representative baseline and ablation variants, including BRICS-based recombination, random SELFIES generation, an LSTM-based SMILES generator, and earlier single-stage pocket-guided versions of the pipeline. Among these tested strategies, the final pocket-guided two-stage GNINA workflow achieved the strongest overall docking performance. In comparison with the BRICS baseline, it improved the mean docking affinity from −8.434 to −8.597, the Top-10 mean from −9.264 to −9.488, and the best docking score from −9.81 to −10.13. These results indicate that the main advantage of our method lies in integrating generative exploration with pocket-aware structural reranking and two-stage docking (Table 1).

**Table 1:** Comprehensive evaluation of docking performance and drug-likeness metrics for the top-ranked generated candidates.

SMILES	Docking_affinity	Docking_CNN_PoseScore	Docking_CNN_Affinity	QED	SA_score	Toxic_alerts
Compound 1	−9.29	0.65	6.178	0.714	3.328	0
Compound 2	−9.09	0.545	7.372	0.414	3.999	0
Compound 3	−8.88	0.712	6.56	0.605	3.293	0

Compounds 1–3 were identified through integrated filtering and docking validation. Detailed interaction residues are shown in Figure 5.

### 3.5 Dynamic stability and binding energetics of compound 1

**Figure 6:**
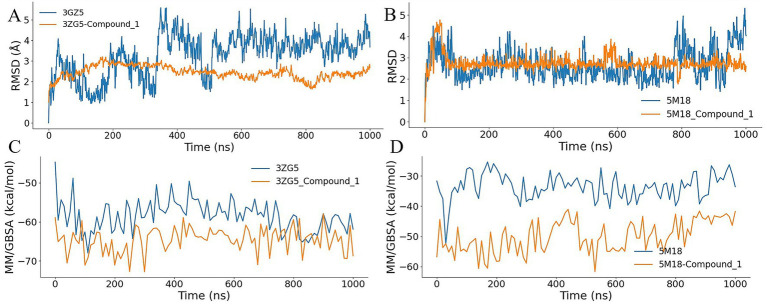
Molecular dynamics simulations and antibacterial activity of Compound 1. **(A,B)** RMSD trajectories over 1,000 ns for Compound 1 and the co-crystal ligands in complex with 3ZG5 and 5 M18, respectively, demonstrating the enhanced stability of the generated lead. **(C,D)** MMGBSA binding free energy comparisons showing the superior affinity of Compound 1 over native inhibitors for both target proteins.

To further evaluate the dynamic stability and binding energetics of the selected lead, 1,000 ns molecular dynamics (MD) simulations were performed on the complexes. As illustrated in Figures 6A,B, the RMSD profiles of Compound 1 in complex with both the 3ZG5 and 5 M18 receptors were compared against those of the respective co-crystal ligands. Throughout the 1,000 ns trajectory, Compound 1 exhibited lower and more consistent RMSD values than the native co-crystal ligands in both protein environments. These results indicate that Compound 1 maintains superior structural stability and resides more securely within the binding pockets than the original inhibitors.

Among the three final lead candidates, Compound 1 was selected for downstream molecular dynamics simulation, MMGBSA binding free-energy analysis, and experimental MIC validation because it showed the most favorable overall combination of docking performance, residue-level interaction consistency, and developability-related property filters. This enhanced stability is further supported by the binding free energy calculations using the MMGBSA method. As shown in Figures 6C,D, Compound 1 consistently demonstrated higher binding affinities for both 3ZG5 and 5 M18 compared to the co-crystal ligands. The combination of lower conformational fluctuations and more favorable energetic profiles across the extended simulation time underscores the robustness of the binding mode identified in the docking phase, reinforcing the potential of Compound 1 as a potent dual-target stabilizer.

### 3.6 Antibacterial activity of compound 1

**Figure 7:**
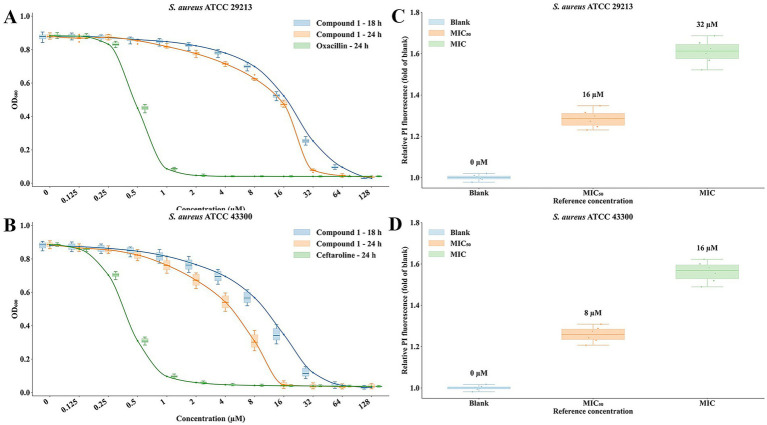
*In vitro* antibacterial activity and PI staining analysis of Compound 1 in *S. aureus* ATCC 29213 and *MRSA* ATCC 43300. **(A)** Dose–response curves of Compound 1 against *S. aureus* ATCC 29213 at 18 h and 24 h. **(B)** Dose–response curves of Compound 1 against *MRSA* ATCC 43300 at 18 h and 24 h, with ceftaroline as a positive control. **(C)** Relative PI fluorescence in *S. aureus* ATCC 29213 after treatment with Compound 1 at blank, MIC_50_ (16 μM), and MIC (32 μM). **(D)** Relative PI fluorescence in *MRSA* ATCC 43300 after treatment with Compound 1 at blank, MIC_50_ (8 μM), and MIC (16 μM). PI fluorescence increased in a concentration-dependent manner in both strains.

The antibacterial activity of Compound 1 was evaluated by broth microdilution against *S. aureus* ATCC 29213 and MRSA ATCC 43300, with bacterial growth quantified by OD600 using six replicates per concentration. In *S. aureus* ATCC 29213 (Figure 7A), Compound 1 exhibited a clear concentration-dependent inhibitory effect at both 18 h and 24 h. At low concentrations (0.125–1 μM), OD_600_ values remained close to the growth control, whereas growth inhibition became progressively more pronounced at 2–16 μM. Notably, the 24 h response curve was left-shifted relative to the 18 h curve, with a marked decline in bacterial growth observed around 16–32 μM and only minimal residual growth at higher concentrations, indicating enhanced time-dependent antibacterial activity.

A similar but more pronounced pattern was observed in *MRSA* ATCC 43300 (Figure 7B). Compound 1 again showed concentration-dependent inhibition at both time points, while the 24 h treatment produced substantially stronger growth suppression than the 18 h treatment. At 18 h, bacterial growth remained largely unaffected at 0.125–2 μM, declined gradually from 4 to 16 μM, and entered a steep inhibitory phase between 32 and 64 μM, yielding an apparent MIC of approximately 64 μM. In contrast, the 24 h curve showed a clear leftward shift, with stronger inhibition emerging at 2–8 μM and OD approaching background levels at 16 μM, corresponding to an apparent MIC of approximately 16 μM. As a positive control, ceftaroline displayed substantially greater potency against *MRSA* ATCC 43300, with a sharp reduction in OD_600_ between 0.25 and 1 μM and an apparent MIC of 1 μM.

To further examine the cellular response to Compound 1, propidium iodide (PI) staining was performed at reference concentrations corresponding to MIC_50_ and MIC in both strains. In *S. aureus* ATCC 29213 (Figure 7C), treatment with Compound 1 increased the relative PI fluorescence from the blank control to approximately 1.28-fold at 16 μM (MIC_50_) and 1.61-fold at 32 μM (MIC), indicating a concentration-dependent increase in PI uptake. A similar trend was observed in *MRSA* ATCC 43300 (Figure 7D), where the relative PI fluorescence increased to approximately 1.26-fold at 8 μM (MIC_50_) and 1.57-fold at 16 μM (MIC) compared with the untreated control. Because PI fluorescence primarily reflects increased membrane permeability or compromised envelope integrity, these results are interpreted as indirect whole-cell evidence of compound-induced envelope perturbation. In the context of the present PBP2a-guided design hypothesis, the observed PI increase may be consistent with a secondary consequence of cell-wall stress, but it does not by itself establish direct target engagement.

To strengthen the biological characterization, we further performed a time-kill analysis together with a preliminary mammalian-cell cytotoxicity assessment in HaCaT cells (Supplementary Figure S1). In *S. aureus* ATCC 29213, treatment with Compound 1 at 32 uM produced a marked time-dependent reduction in relative viable count, decreasing to approximately 20% at 12 h and approaching background levels by 24 h. In *MRSA* ATCC 43300, treatment with 16 uM Compound 1 caused an even more rapid decline, with viable counts reduced to near-background levels by 12 h and remaining strongly suppressed through 36 h. In parallel, HaCaT cells exposed to 16 or 32 uM Compound 1 did not show an obvious loss of viability over the same observation period; instead, relative cell viability remained at or above baseline under the tested conditions. These results provide additional support for the time-dependent antibacterial effect of Compound 1 while suggesting no evident cytotoxicity toward mammalian cells at concentrations associated with antibacterial activity.

Collectively, these results demonstrate that Compound 1 possesses time-dependent antibacterial activity and exhibits enhanced inhibitory efficacy upon prolonged exposure, while remaining less potent than ceftaroline against MRSA. The accompanying PI staining data further support that Compound 1 induces a concentration-dependent whole-cell envelope stress phenotype in both strains.

## 4 Discussion

The global escalation of antimicrobial resistance represents a formidable challenge to modern medicine as MRSA remains a primary cause of high morbidity and mortality worldwide (Patra et al., 2025). This resistance is fundamentally mediated by the expression of PBP2A, a transpeptidase that allows cell wall biosynthesis to persist even in the presence of lethal drug concentrations due to its extremely low affinity for antibiotics (Adedeji-Olulana et al., 2024). Traditional structure-based drug design often fails because it relies on static snapshots from crystallography that do not capture the transient druggable states of this highly flexible protein (Lam and Katritch, 2025; Fraser and Murcko, 2024). Our study addressed these limitations by integrating ensemble-based structural biology with deep generative modeling to identify next-generation inhibitors (Aranganathan et al., 2025; Zhou et al., 2026). By employing extensive MD simulations and enhanced sampling techniques, we mapped the structural plasticity of the target to reveal cryptic pockets and intermediate open configurations that are invisible in static structures (Shen et al., 2023). These ensembles represented a statistical distribution of structural states that allowed for the identification of inhibitors capable of either stabilizing the inactive state or exploiting transiently exposed motifs (Brotzakis et al., 2025; Sala et al., 2023).

The integration of deep learning-based generative models revolutionized the expansion of the accessible chemical space by enabling the *de novo* design of novel molecular scaffolds with optimized pharmacological properties. By conditioning these generative models on the structural features derived from the conformational ensembles, it was possible to tailor molecules that satisfy the complex geometric and electrostatic requirements of dynamic binding sites (Brownless et al., 2025). The evolutionary optimization process navigated a vast chemical space while maintaining a balance between binding affinity, drug-likeness, and synthetic feasibility (Jeon et al., 2025; Xia et al., 2024). Although the evolutionary trajectories exhibited fluctuations in average fitness scores due to stringent penalties for non-compliant candidates, the elite preservation mechanisms successfully and steadily drove the upper bound of molecular quality forward (García-Pintos, 2024). PCA and pairwise Tanimoto similarity analysis confirmed that the generative model successfully navigated into novel chemical territories far beyond the structural neighborhood of the starting seeds, achieving high scaffold variety and internal diversity (Schottlender et al., 2025). Feature importance analysis identified QED and MW as the primary determinants driving the optimization, ensuring that the workflow successfully navigated the trade-off between increasing molecular surface area for binding and managing potential ADMET risks (Chan and Veas, 2024).

Among the prioritized candidates, Compound 1 emerged as the most promising lead because it demonstrated a highly conserved binding mode across multiple complex models, sharing key residues such as ARG-241, ARG-151, VAL-277, and TYR-373. This structural robustness stood in stark contrast to other candidates which exhibited poor residue-level consistency and likely represented less stable binding orientations (Artime et al., 2024). Throughout 1,000 ns MD simulations, Compound 1 maintained superior structural stability and resided more securely within the binding pockets than the original co-crystal inhibitors. This enhanced stability was further supported by binding free energy calculations using the MMGBSA approach, which consistently demonstrated higher affinities for the target protein compared to native ligands (Bhati and Coveney, 2022; Moore et al., 2023; Cournia et al., 2017). The biological efficacy of the selected lead was further supported by whole-cell antibacterial assays against *S. aureus* ATCC 29213 and MRSA ATCC 43300, in which Compound 1 produced dose-dependent growth inhibition with stronger activity after 24 h exposure. In addition, PI staining revealed a concentration-dependent increase in PI uptake in both strains under MIC50 and MIC conditions, consistent with compound-induced envelope perturbation. Within the context of the present PBP2A-guided design hypothesis, this phenotype may reflect a secondary consequence of cell-wall stress; however, it should not be interpreted as direct evidence of PBP2A engagement. Together, these results support Compound 1 as a viable antibacterial lead identified through a generalizable workflow for highly flexible protein targets.

## 5 Conclusion

In summary, this study presents an integrated workflow for PBP2A-guided discovery by coupling conformational ensemble analysis with deep generative molecular design. MD simulations of the 3ZG5 and 5 M18 structures yielded 20 representative receptor states that enabled pocket-aware molecular generation, and the resulting evolutionary search produced a chemically diverse library of 120 unique molecules that moved beyond the local neighborhood of the seed compounds while maintaining developability constraints. Through successive ensemble docking, residue-level interaction consistency analysis, 1,000 ns MD simulations, and MMGBSA calculations, Compound 1 emerged as the most robust lead, showing conserved interactions with ARG-241, ARG-151, VAL-277, and TYR-373 together with greater structural stability and more favorable binding energetics than the native ligands. The computational prioritization was further supported by whole-cell antibacterial testing against *S. aureus* ATCC 29213 and MRSA ATCC 43300, in which Compound 1 showed dose-dependent growth inhibition and enhanced activity after prolonged exposure. PI staining further revealed concentration-dependent increases in PI uptake in both strains, consistent with compound-induced envelope perturbation. Collectively, these findings indicate that explicitly incorporating protein dynamics into generative drug design can improve hit discovery for targets that are poorly represented by static structures and support Compound 1 as an antibacterial lead emerging from a PBP2A-guided discovery strategy; however, direct biochemical or biophysical validation will be required to establish the target-level mechanism.

## Data Availability

The raw data supporting the conclusions of this article will be made available by the authors, without undue reservation.

## Footnotes

1
www.bindingdb.org
