# Supplementary figures and images for: Integrated framework for targeting dynamic penicillin-binding protein 2a via ensemble structural biology and deep generative modeling
Source: Front Microbiol. 2026 May 11;17:1811950. doi: 10.3389/fmicb.2026.1811950 (PMC13199089; doi:10.3389/fmicb.2026.1811950)

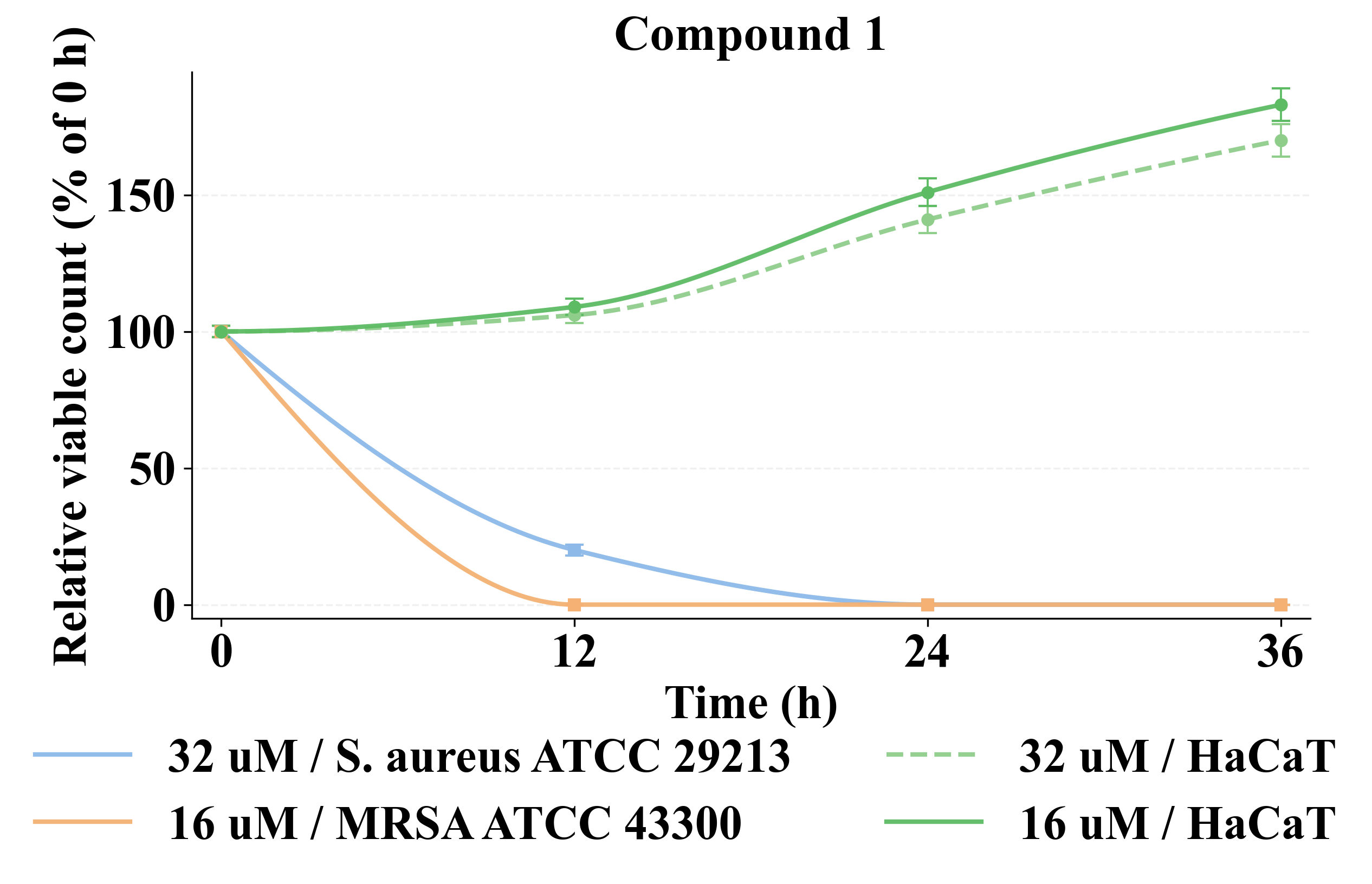

Supplement: Supplementary file 2 [file Image_1.PNG]
